# Identification of DEP domain-containing proteins by a machine learning method and experimental analysis of their expression in human HCC tissues

**DOI:** 10.1038/srep39655

**Published:** 2016-12-21

**Authors:** Zhijun Liao, Xinrui Wang, Yeting Zeng, Quan Zou

**Affiliations:** 1 Department of Biochemistry and Molecular Biology, School of Basic Medical Sciences, Fujian Medical University, Fuzhou 350122, China; 2 School of Computer Science and Technology, Tianjin University, Tianjin 300354, China; 3 State Key Laboratory for Medical Genomics, Shanghai Institute of Hematology, Rui-Jin Hospital affiliated to School of Medicine, Shanghai Jiao Tong University, Shanghai 200025, China; 4 Department of Pathology, Dongfang Hospital, Fuzhou 350025, China; 5 State Key Laboratory of Medicinal Chemical Biology, Nankai University, Tianjin 300074, China

## Abstract

The Dishevelled/EGL-10/Pleckstrin (DEP) domain-containing (DEPDC) proteins have seven members. However, whether this superfamily can be distinguished from other proteins based only on the amino acid sequences, remains unknown. Here, we describe a computational method to segregate DEPDCs and non-DEPDCs. First, we examined the Pfam numbers of the known DEPDCs and used the longest sequences for each Pfam to construct a phylogenetic tree. Subsequently, we extracted 188-dimensional (188D) and 20D features of DEPDCs and non-DEPDCs and classified them with random forest classifier. We also mined the motifs of human DEPDCs to find the related domains. Finally, we designed experimental verification methods of human DEPDC expression at the mRNA level in hepatocellular carcinoma (HCC) and adjacent normal tissues. The phylogenetic analysis showed that the DEPDCs superfamily can be divided into three clusters. Moreover, the 188D and 20D features can both be used to effectively distinguish the two protein types. Motif analysis revealed that the DEP and RhoGAP domain was common in human DEPDCs, human HCC and the adjacent tissues that widely expressed DEPDCs. However, their regulation was not identical. In conclusion, we successfully constructed a binary classifier for DEPDCs and experimentally verified their expression in human HCC tissues.

The Dishevelled (first recognized in *Drosophila*)^1^, EGL-10 (first confirmed in *Caenorhabditis elegans*)^2^ and Pleckstrin (first identified in *mammals*)^3^ (DEP) domain-containing (DEPDC) proteins were discovered to have seven members, namely DEPDC1-DEPDC7. Most of which were found involving in signal transduction^4^,^5^,^6^. The domain is defined as a specific combination of secondary structures organized into a characteristic three-dimensional structure or fold, and generally as a transmembrane region or functional region. Meanwhile, different combinations of domains can generate the diverse range of proteins found in nature. Therefore, the identification of domains that occur within proteins can provide insights into their functions. DEP domains (approximately 80 amino acids) are usually globular protein domains, which may facilitate translocation of the homologous protein to the plasma membrane. The structure of the mouse Dishevelled 1 DEP domain is characterized by a three-helix bundle (H1-H3), a β-hairpin “arm” with two β-strands (B1 and B2) lying between H1 and H2, and two short β-strands (B3 and B4) at the C-terminal region as revealed by nuclear magnetic resonance (Fig. 1)^7^. Three α-helices composed of highly conserved hydrophobic amino acids stabilize the core structure of this domain. The DEP domain is usually located in the residue range of 402-495. Among these areas, Lys434, Asp445 and Asp448 create a strong electric dipole interaction with regulators upstream of Dvl. This interaction is an important mechanism in the transduction of the Wnt signaling pathway.

DEPDC1 is a highly conserved protein that was first found to over-express in bladder cancer cells^8^ in 2007; hence, this protein has been identified as a therapy target^9^. Furthermore, DEPDC1 is related to several types of cancer and contributes to carcinogenesis. For example, DEPDC1 is over-expressed in colorectal cancer^10^ and up-regulated in lung adenocarcinomas^11^, and as one of the 16 genes with concomitant genomic alterations that participate in breast cancer^12^. DEPDC1 could also be involved in the activation of NF-κB cell survival as a transcriptional repressor^13^. One of its members, DEPDC1B coordinated the de-adhesion events along the DEPDC1B/RhoA/PTPRF axis on mitotic dynamics during zebrafish development^14^. Moreover, the expression of DEPDC1B, which is a potential Rho GTPase-activating protein correlated to oral cancer^6^, might be repressed by Pitx2 when contributing to signaling pathways^15^. Yuan^16^ discovered the high DEPDC1 expression in hepatocellular carcinoma (HCC) tissues at the mRNA and protein levels. Given the higher DEPDC1 expression in HCC patients, the overall survival and disease free survival rate become poorer. In response to the anti-tubulins in *C. elegans*, MCL1 (a member of Bcl-2 family) occurrs via an evolutionarily conserved signaling pathway that involves the DEPDC protein LET-99 (DEPDC1 homolog in mammals). This concept suggests that DEPDC1 participates in the anti-tubulin drug-induced apoptotic cell death pathway^17^,^18^. Therefore, DEPDC1 is identified as a novel tumor-related gene^19^.

DEPDC2 is broadly expressed in human and zebrafish^20^, and it is a candidate molecular marker for human embryonic stem cells^21^. Three transcription factors (OCT4, SOX2, and NANOG) are required for the transcriptional regulation of DEPDC2^22^, which may be a candidate gene linked to febrile seizures of epilepsy^23^. DEPDC2 is also called the phosphatidylinositol-3,4,5-trisphosphate-dependent Rac exchange factor 2 (PREX2), which is a Rac guanine nucleotide exchange factor (Rac-GEF). Previous reports have stated that PREX2 is over-expressed in human HCCs^24^,^25^ and melanomas; PREX2 also acts as a PTEN binding and inhibiting protein^26^,^27^. Although some studies have revealed limited information of DEPDC2, its exact function remains largely unknown.

DEPDC3 has no related literature in PubMed, but it is also known as the G-protein-coupled receptor 155 (Gpr155), which is conserved among mammals and may be a candidate gene for type 2 diabetes in mouse models^28^. As a 17-transmembrane (TM) protein, Gpr155 is widely expressed in adult mouse tissues, and it has a carrier domain, a G-protein-interacting region and a DNA-binding domain^29^. Gpr155 might also play an important role in the GABAergic neurotransmission involved in sensory information processing and memory^30^. However, specific information on Gpr155 protein function has not been elucidated.

DEPDC4 is also a signaling molecule involved in G-protein-coupled receptor (GPCR) signaling pathways. The gene sequences of this protein were disrupted by rearrangement in the gibbon genome during mapping around the breakpoint regions, as compared with the human genome^31^. Hawthorne^32^ reported that one single nucleotide polymorphism (SNP) in its 3′-untranslated region was associated with the high-grade myopia MYP3 gene. However, the function of DEPDC4 remains largely unknown.

DEPDC5 is a protein that is strongly associated with various of familial epilepsies^33^, such as familial focal epilepsies^34^, familial temporal lobe epilepsy^35^, and autosomal dominant nocturnal frontal lobe epilepsy^36^. Pippucci^37^ discovered that two different truncating mutations of the DEPDC5 gene are involved in uncommon presented focal epilepsy with auditory features based on whole-exome sequencing. Several DEPDC5-related hereditary mutations are correlated with focal epileptic spasms in a cohort of patients and controls. In addition, DEPDC5 variation might be the most frequent gene in epileptic spasms; its variants are associated with focal cortical dysplasia (FCD) type IIA^38^. As an important anti-rapamycin regulatory gene, DEPDC5 mutations play an inhibitory role in the malformations of cortical development (MCD) of epilepsy. Moreover, DEPDC5 is also a signaling molecule involved in the phosphoinositide 3-kinase (PI3K)-AKT-mTOR pathway and an important component of the GAP activity on RAGs complex1 (GATOR1), which is a negative regulator of mTOR. Research on SNPs suggested that DEPDC5 polymorphisms affect the progression of HBV-related liver disease^39^. Miki^40^ identified an intronic SNP variant of DEPDC5 in a Japanese patient with chronic HCV-related HCC. However, when HCV-positive HCC recurrence patients who have undergone hepatectomy, no correlations were found between the DEPDC5 genetic polymorphism and the recurring patients^41^.

DEPDC6 is also known as the DEPDC mTOR-interacting (DEPTOR) protein, which can only be found in vertebrates. mTOR is a conserved serine/threonine-protein kinase forming mTOR complex [mTORC] 1 and mTORC2^42^. As an important member of mTOR complex, human DEPTOR contains two N-terminal DEP domains and one C-terminal PDZ domain. In addition, DEPTOR is an endogenous inhibitor of both mTORCs via its PDZ domain^43^. Therefore, DEPTOR is involved in the mTOR-dependent signaling pathway; specifically, DEPTOR inhibits the mTORC1/PI3K pathway and activats Akt, whereas mTOR is a strongly negative regulator of autophagy, which is correlated with diabetes mellitus and energy metabolism^44^. The DEPTOR-mTOR signaling pathway was regulated by glutamine administration in colitis-associated colorectal cancer mice^45^. Moreover, DEPTOR binding weakened for both mTOR complexes when mutations in the FRAP-ATM-TTRAP (FAT) domain in clear cell renal cell carcinoma. Consequently, point mutations in the FAT domain promoted mTORC1 and mTORC2 activity, as well as increased cancer cell proliferation, thereby decreasing DEPTOR binding and indicating poor patient prognosis^46^. Abnormally high DEPTOR expression activated the PI3K-AKT pathway and was regarded as a poor prognostic biomarker, which has been found in various types of solid neoplasms such as esophageal squamous cell carcinoma^47^, breast cancer^48^,^49^, and HCC^50^. However, DEPTOR was also reported playing dichotomous functions of the proliferation and metastasis in breast cancer^51^.

Three years ago, DEPDC7 was called LOC91614 and relatively unknown; however, our group has given close attention to this gene and studied it for several years. Our previous studies showed the high level of DEPDC7 differential expression in HCC tissues and hepatoma cell lines; the gene is closely correlated with the proliferation, migration, and invasion capacity as verified by RNA interference researches^52^. Furthermore, DEPDC7 can interact with CARMA2 and CARMA3 proteins as a positive regulator as well as active NF-κB signal transduction pathway^5^. A recent report indicated that DEPDC7 DNA intron hypomethylation may be correlated with depression^53^, DEPDC7 deletion may also be one factor of azoospermia in cryptorchidism patients, thereby implying its influence on reproduction^54^. Consequently, further studies on DEPDC7 are needed.

Machine learning, developed from computational learning and pattern recognition theory, makes computers able to learn without being explicitly programmed, and it has been widely used to devise prediction models^55^. Classification machine learning models can be validated by many accuracy estimation methods and evaluated by tools for classification model assessment. Random Forest(RF) is an ensemble classifier that has been proven to be robust in classification issue with high dimensional data, which is often employed in handling bioinformatics problems^56^. Although the class predictions are averaged multiple deep decision RF trees, the final model prediction is based on the majority vote^57^.

Based on the sequence and other physicochemical properties of each protein, we discriminated and predicted the DEPDC proteins from non-DEPDC proteins with a machine learning algorithm by extracting the 188D and 20D feature vectors and constructing a binary classifier for this purpose in this study. Subsequently, we searched for the main motifs of human DEPDC proteins, which are related to their functional domains. Finally, we performed experimental verification of human DEPDC gene expression with qRT-PCR in HCC and adjacent normal tissues.

## Results

### Phylogenetic analysis of positive Pfam corresponding longest protein sequences

**Figure 2:**
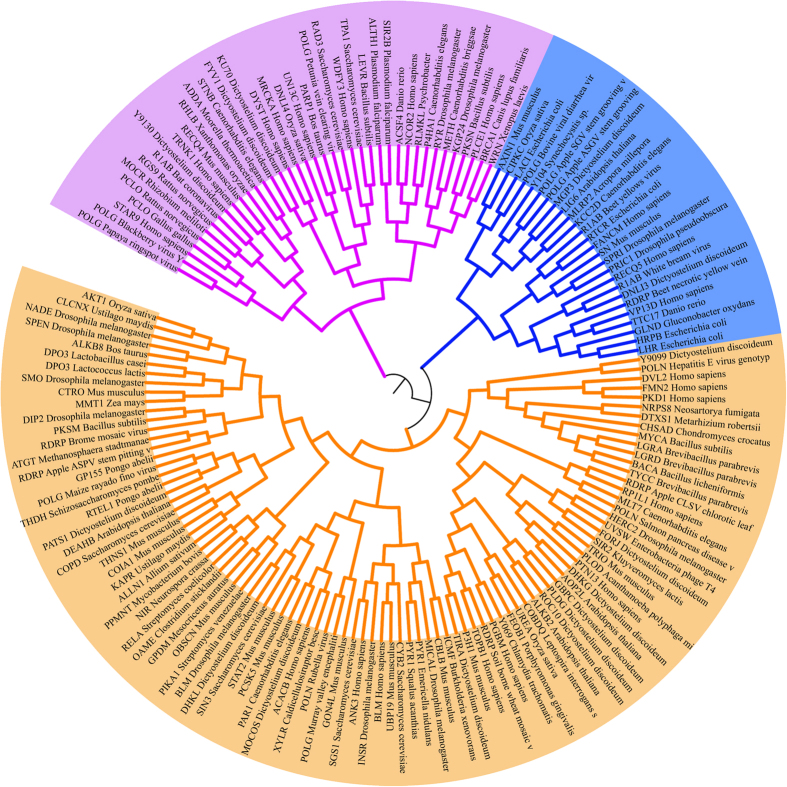
Rooted neighbor-joining tree based on p-distance in a circular polar form as reconstructed from 160 full-length sequences of the positive Pfam family.

Based on the neighbor-joining algorithm and bootstrap method for phylogeny test, the number of bootstrap replicates was set to 500, and the tree was out-grouped with the “Root On Midpoint” option^58^. Subsequently, we built a robust circular polar phylogenetic tree of the whole positive 160 Pfam-containing sequence members (Fig. 2). The general presentation of this tree can obviously distinguish all members into three main classes, Cluster I–III, which included a total of 71 species. From the figure, the Cluster Icontained 45 species (right), Cluster II kept 24 species (bottom left), and Cluster III had 19 species (upper left). Among these clusters, the 21 human proteins were distributed between Clusters I and II.

### Reclassification of positive and negative proteins

**Figure 3:**
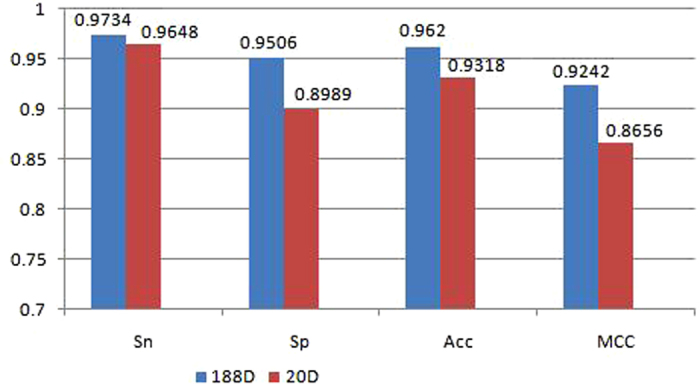
*Sn, Sp, Acc*, and *MCC* values listed for 188D and 20D methods.

We obtained data on the 188D and 20D feature vectors from the positive and negative groups for import into the Weka explorer. The results showed that the correctly classified rates were 96.2% and 93.2%, whereas the ROC area reached 0.995 and 0.983. The confusion matrix is shown as Table 1. The four common measured features are illustrated in Fig. 3.

### Analysis of human DEPDC proteins for phylogenetics and conserved motif composition

**Figure 4:**
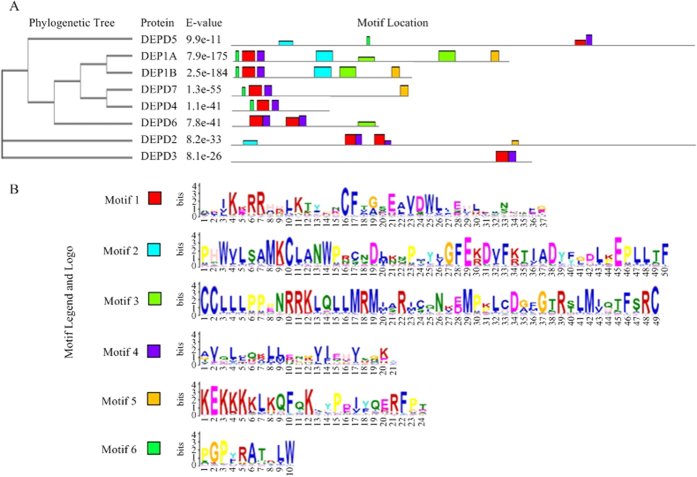
Motifs of human DEPDC proteins as found by the MEME/MAST system. (**A**) Eight human DEPDC protein sequences were initially used for MSA to construct a phylogenetic tree with the MAFFT program before searching for the motifs with the MEME/MAST software. (**B**) The corresponding six-motif legends and logos as visualized for human DEPDC proteins (details in Table 2).

Our phylogenetic analyses with MAFFT revealed that the eight human DEPDC members can be divided into three main groups (Fig. 4 and Table 2). Group I includes DEPDC5, DEPDC1A, DEPDC1B, DEPDC7, DEPDC4, and DEPDC6. Group II includes DEPDC2 only. Group III includes DEPDC3 only. Thus, the six members of group I possess the more sequence similarities than the other two groups. To a certain extent, this indicated that Group I proteins shared evolutionary origins. Among the six searched motifs, Motif 1 and 4 comprise the DEP domain in all members, whereas Motif 2 and 3 are part of the GTPase-activator protein for Rho-like small GTPases (RhoGAP) domain located near the C-terminus. Small GTPases act as molecular switches to control the active or inactive effectors.

### Human DEPDC proteins were expressed in HCC and their paired adjacent tissues

**Figure 5:**
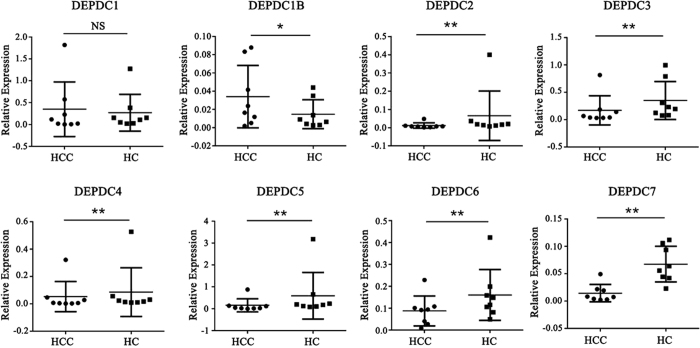
Expression of human DEPDC genes in HCC samples compared with paired adjacent normal tissues (HC) presented as mean ± standard error. *P < 0.05, **P < 0.01 (nonparametric Mann–Whitney U-test). NS, not significant.

Human DEPDCs were broadly expressed in HCC. Most genes showed significant differential expression (Fig. 5). The expression level of DEPDC genes in HCC samples and paired adjacent normal tissues (HC) were analyzed with quantitative real-time PCR (qPCR). As shown in Fig. 5, DEPDC1B was up-regulated as compared with HC. The other six genes were down-regulated, with the exception of DEPDC1 whose difference was not significant. The combined data suggest the significant variations among DEPDC family genes.

## Discussion

The DEPDC protein family includes seven members, which contain the DEP domain as their common characteristic; these proteins can mediate protein-to-protein interaction and membrane targeting of signal molecules^5^,^18^,^59^,^60^. Consequently, most DEPDCs are involved in signal transduction and closely related to several other signal molecules. For instance, these molecules participate in the Wnt^61^, mTOR, NF-κB, PI3K-AKT^4^, and G-protein signaling pathways^62^. Besides the DEP domain, some DEPDCs contain other domains such as PDZ and GAP, which play various roles in protein-to-protein interaction.

In this study, we performed binary-class classification of the DEPDC and non-DEPDC family. First, a phylogenetic tree was built from all the longest positive Pfam-containing sequences and discovered that the DEPDC family can be divided into three clusters. Based on protein sequence and isual-chemical characteristics, we extracted the 188D and 20D feature vectors on the positive and negative datasets by machine learning, as predicted by RF classifier. We obtained relatively high effects on the correctly classified rates and the AUC value. Similarly, the Sn, Sp, Acc, and MCC values reached relatively superior results. To the best of our knowledge, we are the first to categorize the DEPDC family; thus, no corresponding pre-existing data is available for comparison. Third, the MEME Suite is suitable for screening common sequence motifs from a set of amino acid sequences^63^. Thus, we applied this technique to analyze the main motifs of human DEPDCs and found that these proteins can be divided into three groups by phylogenetic analyses. Group I contains six of the eight DEPDC members, thereby suggesting their closer relationship and evolutionary origin. On the other hand, some motifs comprise the DEP and RhoGAP domains.

Finally, we also used experimental methods to verify the gene expression of the human DEPDC family in HCC and adjacent normal tissues. From the qRT-PCR results, we observed that human DEPDCs were widely expressed in the cancer tissue. However, direction of the expression was not identical. Our experiment showed that DEPDC1 expression was not affected between the two tissues. DEPDC1B was up-regulated in HCC, whereas the other six genes (DEPDC2- DEPDC7) were down-regulated in HCC. This trend may indicate their different functional correlation and great variation. Further study on the regulation and function of these genes with HCC or other related cancers is needed.

In conclusion, we have successfully constructed a binary-class model algorithm to split the DEPDCs with non-DEPDCs, and the 160 positive Pfam-containing members can be differentiated into three main clusters. The eight human DEPDCs can be divided into three groups from molecular phylogenetic analyses and the conserved DEP and RhoGAP domains were discovered by MEME tools. Finally, we verified the eight human DEPDCs mRNA expression with experiment in human HCC tissues.

## Methods

### Data retrieval and treatment

The primary sequences of DEPDC proteins and the control Pfam proteins (as FASTA files) were retrieved from the Universal Protein Resource (UniProt) database (www.uniprot.org)^64^. To reduce the sequence redundancy and improve analytical performance, the raw data were preprocessed by the CD-HIT program (http://cd-hit.org) for merging the sequence similarities^65^, which has been widely used in bioinformatics^66^. In the present study, the sequence identity cut-off was set at 0.90, whereas the other default parameters were used to avoid bias during categorization. We obtained 1416 DEPDC sequences for the positive dataset; the negative samples were identified from the control proteins after removing the positive samples. Finally, we acquired 10585 entries as negative dataset.

### Multiple sequence alignment (MSA) and phylogenetic tree construction based on positive Pfam

All positive DEPDCs were applied to extract their corresponding protein families (returned Pfam number) from the Uniprot “Family & Domains” section. After excluding the identical and redundant entries, we acquired 229 unique Pfam numbers (the names begin with PF). To construct a phylogenetic tree^67^,^68^,^69^, we initially extracted the longest sequence from each Pfam containing member of the positive dataset (in.fasta format) and identified 160 sequences by combining the same records. Second, these sequences were subjected to perform MSA with default parameters of CLUSTAL X2.1^70^. We performed the “Do Complete Alignment” and provided the resulting alignments in an.aln CLUSTAL file as output. Finally, the.aln file was transformed into a.meg format for the MEGA 6 program^71^ to construct a neighbor-joining tree with p-distance model. Other default parameters were kept, whereas phylogenetic analysis was isualized in a circular polar tree.

### Prediction analyses of DEPDCs

To fully uncover the important information hidden in protein sequences, we constructed two feature extraction algorithms: the 188-dimensional(188D) and the simplified 20D feature vectors. First, we extracted the feature vectors from the positive and negative protein sequence dataset by a previously developed novel machine learning method^72^,^73^,^74^. We transformed all the positive and negative datasets into the corresponding protein family information (Pfam number files). Meanwhile, the sequence evolutional information, the k-skip-n-gram model, physicochemical properties, local PsePSSM, and other features were obtained^75^. Subsequently, we extracted the longest sequence from both datasets of each Pfam protein family(as.fasta files). Finally, the.fasta files were assembled into 188D and 20D feature vectors. A detailed description of the methods are shown in refs 72 and 76.

After the above mentioned process, the resulting feature vectors are imported into weka (http://www.cs.waikato.ac.nz/ml/weka/), which is a machine learning workbench. In weka, we filtered the vector data with the synthetic minority over-sampling technique(SMOTE)^77^,^78^,^79^ and changed the positive instances from the 100% into 700% to overcome the highly imbalanced data. the vector data were automatically classified by visualization and cross-validation analysis^80^,^81^,^82^,^83^,^84^,^85^. Based on the optimal features in some preliminary trials on the same dataset, we finally selected RF module to distinguish the two classes and utilize the ten-fold cross-validation model.

To measure the performance quality of the statistical classification more intuitively in machine learning, we calculated four common parameters for evaluating the classifier: sensitivity (*Sn*), specificity (*Sp*), accuracy (*Acc*) and Matthew’s correlation coefficient (*MCC)*. The formulas of these parameters are^86^,^87^,^88^,^89^:


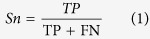



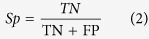



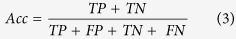



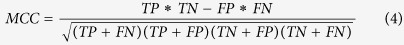


where *TP(true positive)* indicates the number of true DEPDCs that are correctly predicted, *TN(true negative)* indicates the number of true non-DEPDCs that are correctly predicted, *FP(false positive)* is the number of true non-DEPDCs that are predicted to be DEPDCs, and *FN(false negative)* is the number of true DEPDCs that are predicted to be non-DEPDCs.

### Conserved motif composition analysis of human DEPDCs

Before motif searching, the eight human DEPDCs (DEPDC1A, DEPDC1B, DEPDC2-7) were implemented by MSA and forwarded to the MAFFT server (http://www.ebi.ac.uk/Tools/msa/mafft/) to generate a phylogenetic tree. MAFFT has been significantly improved in accuracy and reduced CPU time as compared with CLUSTALW; the algorithm is also faster than T-COFFEE^90^,^91^. The default parameters were kept unchanged. The MEME Suite (http://meme-suite.org/, 4.11.2 version) was performed for conserved motif analysis. A motif can be assumed as a conserved sequence pattern that repeatedly occurs in a group of related sequences. MEME is a powerful motif-based sequence analysis suite, which can promptly discover novel, ungapped motifs by integrating various sequence analysis tools for proteins, DNA and RNA^92^. The maximum motif number was set to 6 and the remaining parameters were set as default values.

### Gene expression analysis for experimental verification with quantitative real-time PCR in human tissues Patients and samples

Pathological sections were obtained from 8 patients with HCC at the Fuzhou Dongfang Hospital in 2015. The pathology slides and institutional pathology reports were reviewed by the pathologists by following the evidence-based practice guidelines in the standardized pathological diagnosis of primary liver cancer.

### Gene expression analysis with quantitative real-time PCR

The primers were designed by the PrimerQuest tool of IDT, which is freely available at https://sg.idtdna.com/primerquest/Home/Index. A BLAST search of the sequences was used to assure that only the selected gene were targeted. The gene and sequence information of the primers are presented in Table 3. The sequences were synthesized by Sangon Biotech. The total RNA was isolated from formalin-fixed paraffin-embedded (FFPE) samples with the FFPE RNA Purification Kit (AmoyDx, China), according to the manufacturer’s instructions. cDNA synthesis was performed on 5 μg of RNA in a 100 μL sample volume with the PrimeScript^TM^ RT reagent Kit (Takara), as recommended by the manufacturer. Real-time PCR was performed on the Step-One^TM^ Real-Time PCR system (Applied Biosystems) with the SYBER Green qPCR Supermix (Roche) under universal thermal cycling parameters (95 °C for 30 sec, 40 cycles of 30 sec at 95 °C and 5 sec at 60 °C). The comparative Ct method was used to quantify gene expression^93^. The target gene expression level was normalized to the expression of the housekeeping gene β-actin within the same sample (−ΔCt), where the relative expression of each gene was calculated with 2^−ΔCt^.

### Statistical analysis

The nonparametric Mann–Whitney U-test^94^ was applied for the statistical comparison of normal and cancerous tissues. The significance level was P < 0.05.

### Ethical Statements

The study is approved by the ethics committee of the Fuzhou Dongfang Hospital and the experimentation is conducted in accordance with the Declaration of Helsinki and Good Clinical Practice (GCP).

## Additional Information

**How to cite this article**: Liao, Z. *et al*. Identification of DEP domain-containing proteins by a machine learning method and experimental analysis of their expression in human HCC tissues. *Sci. Rep.* **6**, 39655; doi: 10.1038/srep39655 (2016).

**Publisher's note:** Springer Nature remains neutral with regard to jurisdictional claims in published maps and institutional affiliations.

## Figures and Tables

**Figure 1:**
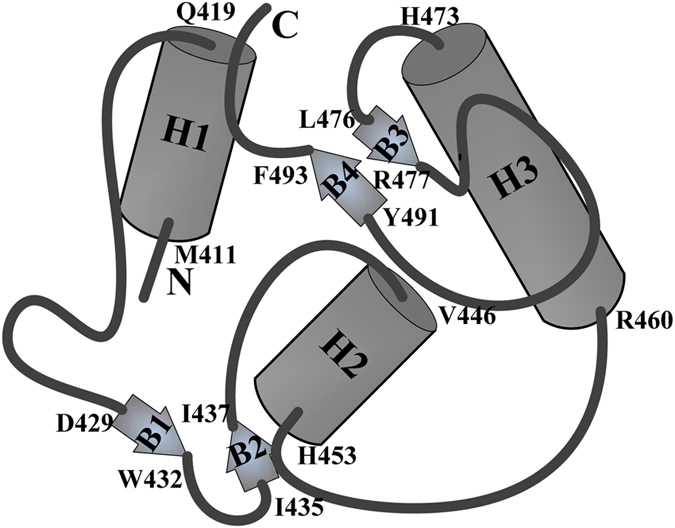
Molecular model of mouse Dishevelled 1 DEP domain structure with the lowest target function. The diagram shows the residue range from the N-terminal 402aa to C-terminal 495aa, including three α helices(H1, H2, and H3) and four β sheets (B1–B4). The numbers in the figure indicate the beginning and end of the corresponding secondary structures. Many hydrophobic residues were situated in H1, H2, H3, B3, B4 to construct the hydrophobic core of DEP domain. Human Dishevelled 1 DEP domain possesses the same structure except for the amino acid position.

**Table 1:** Confusion matrix from RF classifier with 188D and 20D methods.

Cases	DEP domain containing proteins(188D)	Non DEP domain containing proteins(188D)	DEP domain containing proteins(20D)	Non DEP domain containing proteins(20D)
Positive cases	11027	523	10929	1070
Negative cases	301	10062	399	9515

**Table 2:** Conserved motifs of human DEPDC proteins identified by the MEME system.

Motif	Width	Best possible match
1	37	MPIKKRRHHLKTYPNCFTGSEAVDWLYEHLMANDNFG
2	50	PHWVLSAMKCLANWPRCNDMNNPMYVGFEKDVFKTIADYFGDLPEPLLTF
3	49	CCLLLPPPNRRKLQLLMRMMARMCQNKDMPPLCDGFGTRTLMIQTFSRC
4	21	AVQLCQKLMEHHVIEHVTGKW
5	24	KEKKKKLKQFQKCYPDIYQERFPT
6	10	PGPYRATCLW

**Table 3:** Human DEPDC and internal control genes and their primer sequences information.

Gene	Primer sequences	Tm(°C)	%GC	Product(bp)
DEPDC1-F	GAAGCAGTGGATTGGCTTTATG	62	45.5	136
DEPDC1-R	CCCACCTCCCTTTGATATCTTC	62	50.0	
DEPDC1B-F	GGAAATTCTGAAAGTCCCTTTGG	62	43.5	98
DEPDC1B-R	CCATATCAGCTCCTGGGTATTT	62	45.5	
DEPDC2-F	GAGCACAAAGCCAAGAGAGA	62	50.0	100
DEPDC2-R	TCCTACAGCATGCACAACAG	62	50.0	
DEPDC3-F	GCAGAGAAATGGTGGAACTCT	62	47.6	105
DEPDC3-R	CTCCTGGTGCTACAGGAAATAC	62	50.0	
DEPDC4-F	GAACCGTAGAGATGGCTTCTG	62	52.4	101
DEPDC4-R	GGGCCTGAAGAGAGTGAATAAT	62	45.5	
DEPDC5-F	CTCCTGTGGCTTCTTGTTAGT	62	50.0	106
DEPDC5-R	TGATGTTGAGTGGGATGAAGAG	62	50.0	
DEPDC6-F	TTGTGGTGCGAGGAAGTAAG	62	47.6	107
DEPDC6-R	CCGTTGACAGAGACGACAAA	62	45.5	
DEPDC7-F	ACCTTCCACTTCTTGACTCCTTAC	57.8	45.8	155
DEPDC7-R	CGAGAGCCACTCATCTTCCTG	57.5	57.1	
β-actin-F	CGTGCGTGACATTAAGGAGAAG	57.2	50.0	176
β-actin-R	GGAAGGAAGGCTGGAAGAGTG	57.5	57.1	

Note: F, forward; R, reverse.
